# Comparison of the Respiratory Toxicity and Total Cholinesterase Activities in Dimethyl Versus Diethyl Paraoxon-Poisoned Rats

**DOI:** 10.3390/toxics7020023

**Published:** 2019-04-16

**Authors:** Pascal Houzé, Alice Hutin, Marc Lejay, Frédéric J. Baud

**Affiliations:** 1 Laboratoire de Biochimie, Hôpital Universitaire Necker-Enfants Malades, Assistance Publique-Hôpitaux de Paris (AP-HP), 75015 Paris, France; 2 Unité de Technologies Chimiques et Biologiques pour la Santé (UTCBS), CNRS UMR8258-U1022, Faculté de Pharmacie Paris Descartes, Université Paris Descartes, 75006 Paris, France; 3 Département d’Anesthésie–Réanimation-SAMU de Paris, Hôpital Universitaire Necker-Enfants Malades, Assistance Publique-Hôpitaux de Paris (AP-HP), 75015 Paris, France; alice.hutin@aphp.fr (A.H.); marc.lejay@aphp.fr (M.L.); frederic.baud@wanadoo.fr (F.J.B.); 4 EA7323 Evaluation of Therapeutics and Pharmacology in Perinatality and Pediatrics-Hôpitaux Universitaires Cochin–Broca–Hôtel Dieu, Site Tarnier, Université Paris Descartes, 75006 Paris, France; 5 Université Paris Diderot, 75013 Paris, France

**Keywords:** dimethyl paraoxon, diethyl paraoxon, rats, plethysmography, respiratory toxicity, cholinesterases

## Abstract

The chemical structure of organophosphate compounds (OPs) is a well-known factor which modifies the acute toxicity of these compounds. We compared ventilation at rest and cholinesterase activities in male Sprague-Dawley rats poisoned with dimethyl paraoxon (DMPO) and diethyl paraoxon (DEPO) at a subcutaneous dose corresponding to 50% of the median lethal dose (MLD). Ventilation at rest was recorded by whole body plethysmography. Total cholinesterase activities were determined by radiometric assay. Both organophosphates decreased significantly the respiratory rate, resulting from an increase in expiratory time. Dimethyl-induced respiratory toxicity spontaneously reversed within 120 min post-injection. Diethyl-induced respiratory toxicity was long-lasting, more than 180 min post-injection. Both organophosphates decreased cholinesterase activities from 10 to 180 min post-injection with the same degree of inhibition of total cholinesterase within an onset at the same times after injection. There were no significant differences in residual cholinesterase activities between dimethyl and diethyl paraoxon groups at any time. The structure of the alkoxy-group is a determinant factor of the late phase of poisoning, conditioning duration of toxicity without significant effects on the magnitude of alteration of respiratory parameters. For same duration and magnitude of cholinesterase inhibition, there was a strong discrepancy in the time-course of effects between the two compounds.

## 1. Introduction

Organophosphate (OP) compounds are used primarily as pesticides in agricultural settings but have also been developed into chemical weapons including nerve agents. Organophosphates account for the majority of pesticide-related unintentional or intentional poisonings in lower- and middle-income countries [1]. However, there is a wide estimation of the number of deaths related to OP poisonings. Over a short period of recent time, values were 100,000 [2], 260,000 [3], and 300,000 deaths per year [4].

Respiratory failure is still considered the primary cause of death in acute organophosphate poisonings [5]. However, Eddleston et al. [6] also outlined a possible role for solvents in the toxicity of agricultural organophosphorus pesticides. The mechanism of organophosphate-induced respiratory failure remains poorly investigated. Acute respiratory failure induced by organophosphates is thought to result from a direct depressant effect on the respiratory center in the brainstem, constriction of and increased secretion by the airways, and paralysis of the respiratory musculature [7,8].

Organophosphate insecticides encompass a wide range of chemical structures. In the nomenclature and structure of organophosphates, the alkoxy groups can be similar or dissimilar but always a methyl or ethyl radical. In contrast, there is a wide variety in the structure of the leaving group [9]. Noteworthy, dimethoxy-organophosphates were reported to have half-time for aging in the range from 2 to 9 h compared to more than 36 h in the case of diethoxy-organophosphates [10]. However, with dimethoxy-organophosphates, the most important process is spontaneous reactivation by hydrolysis of the dimethoxy-phosphorylated structure unless substantial aging has occurred. As outlined by Marrs [9], the clinical effects on the autonomic nervous system and the neuromuscular junction depend on the balance between on one hand the rate of reaction of organophosphates with cholinesterases, and on the other hand the rate of reactivation and aging of the resultant complex.

**Figure 1:**
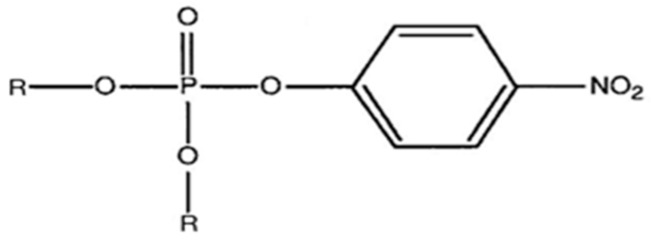
Comparative structure of two derivatives from paraoxon. R = -CH_3_ for dimethyl paraoxon (DMPO) and R= -CH_2_-CH_3_ for diethyl paraoxon (DEPO).

To investigate the complex relationship between chemical structure and respiratory toxicity, we compared dimethyl paraoxon (DMPO) versus diethyl paraoxon (DEPO) (Figure 1) at equipotent non-lethal dose, using whole-body plethysmography in awake rats.

## 2. Materials and Methods

All animal procedures used in this study were approved (identification code: P2.FB.148.10, date of approval: 9 September 2010) by the Regional Ethical Committee of Animal Experimentation (Paris Descartes University).

### 2.1. Animals

Male Sprague-Dawley rats (200 and 250g) were purchased from JANVIER (Le Genest-Saint-Isle, France). Animals were housed in a light- and temperature- controlled setting with access to food and water ad libitum. After each experiment, rats were euthanized using an overdose of pentobarbital.

### 2.2. Chemicals and Drugs

DEPO (diethyl p-nitrophenyl phosphate, CAS number 311-45-5, purity higher than 90%) and DMPO (dimethyl p-nitrophenyl phosphate, CAS number 950-35-6, purity 96%) were obtained from Sigma-Aldrich (Saint-Quentin-Fallavier, France). DEPO solutions were prepared as previously reported [11]. Briefly, DEPO was diluted in dimethyl sulfoxide to obtain a mother solution of 1.0 mg/mL. A daughter solution was prepared extemporaneously in isotonic saline solution (140 µg/mL) to facilitate the injection of doses equal to 50% of the median lethal dose (MLD). DMPO was diluted in dimethyl sulfoxide to obtain a mother solution of 0.96 mg/mL. A daughter solution was prepared extemporaneously in isotonic saline solution (192 µg/mL) to facilitate the injection of doses equal to 50% of the MLD. All solutions of DEPO and DMPO were preserved at 4 °C in the dark for a maximum period of four weeks.

Dimethyl sulfoxide, acetylcholinesterase of the electric eel *Electrophorus electricus* (AchE; 200 UI/mg), dehydrated monosodium phosphate, dehydrated disodium phosphate, chloroacetic acid, hydrochloric acid, sodium chloride, and sodium hydroxide were obtained from Sigma-Aldrich (Saint-Quentin-Fallavier, France). Heparine solution (Choay ™) at 25,000 U.I./5 mL was obtained from Sanofi Winthrop (Gentilly, France). [3H]Acetylcholine iodide (specific activity, 100 Ci/mmol) was obtained from PerkinElmer (Courtaboeuf, France) and was stored at −80°C. Acetylcholine iodide (mw: 273 g/mol, C7H16INO2) was obtained from Fluka (Paris, France) and was stored at −80 °C. Distilled water (Frésenius FrancePharma, Louviers, France) was used for preparation of the various reagents.

### 2.3. Safety Precautions

All solutions of DMPO and DEPO were prepared under fume hood using nitrile gloves, overalls, and goggles, as previously reported [11].

### 2.4. Methods

#### 2.4.1. Clinical Examination and Central Temperature Measurement

The animals were clinically observed while plethysmography measurements were performed. The signs were noted and quantified according to De Candole et al. [12]. Clinical examination was semi-quantitative and realized by two experimenters, as previously reported [8,13]. The central core temperature was simultaneously measured by infra-red telemetry [12,13].

#### 2.4.2. Determination of the Median Lethal Doses (MLDs)

Every effort was made to reduce the number of animals required for the study. Accordingly, the up-and-down method as previously reported by Bruce and refined by Dixon [14,15] was used. DMPO was administered by the subcutaneous route. The MLD dose of only DMPO was determined in this study. We previously determined the MLD dose (0.215 mg/kg) of DEPO [11].

#### 2.4.3. Whole Body Plethysmography

Respiratory parameters were recorded in whole-body plethysmography using the barometric method validated in the rat by Bartlett and Tenney [16] and described in our previous studies with minor modifications [8,11,13,17]. The following parameters were measured: barometric pressure, chamber temperature, core temperature, respiratory frequency (ƒ), inspiratory time (TI), expiratory time (TE), total time (TTOT = TI + TE), tidal volume (VT), and minute ventilation (VE = VT × ƒ).

#### 2.4.4. Measurement of Whole Blood Cholinesterase Activities

##### Blood Samplings

Blood collection was made by tail punctures in heparinized capillaries. All blood sampling was collected in heparinized Eppendorf cups and stored at −80 °C until the assay.

##### Radiometric Assay

Total cholinesterase (butyrylcholinesterase and acetylcholinesterase) activity was measured radiometrically by the method presented by Johnson and Russell [18] using [3H] acetylcholine iodide as substrate, and previously reported by Duarte et al. [19]. Cholinesterase activities measured in whole blood were reported as cpm/min per/mL of erythrocytes. Cholinesterase activities were expressed as the percent of control activity and plotted as a function of time after treatment [19].

### 2.5. Study Designs

Animals were randomized with regard to drug administration in non-blinded study designs. At the end of the experiments, the rats were euthanized with an overdose injection of sodium pentobarbital solution.

#### 2.5.1. Study 1: Determination of the Median Lethal Doses (MLDs) of DMPO in Rats

DMPO was administered in awake, unrestrained animals, via subcutaneous injection in the neck at a 0.50 mg/kg dose corresponding to the LD50 reported in the literature [20]. The method was the same as that previously reported to determine the MLD of DEPO [11].

#### 2.5.2. Study 2: Rats’ Core Temperature Measurement

Core temperature was studied in three groups of six animals. Measurements were performed at the recording times of plethysmography (T5, T10, T15, T20, T30, T45, T60, T90, T120, T150, T180 min) to establish temperature profiles in the control (*n* = 6) and DMPO (*n* = 6) and DEPO groups (*n* = 6). The control group received the solvent of paraoxon compounds (isotonic saline solution, SC). The treated groups received DMPO and DEPO at 50% of MLD (SC). Temperature values were used, at each time, for calculating respiratory parameters in the plethysmography study.

#### 2.5.3. Study 3: Plethysmography Study of the Effects of DEMO and DEPO on Ventilation at Rest in Rats

Ventilation at rest was studied in the same three groups as the study 2. The control group received the solvent of DEPO or DMPO (isotonic saline solution, SC). One treated group received DEPO at 0.215 mg/kg corresponding to 50% of the MLD (SC). The second group received DMPO at 0.250 mg/kg corresponding to 50% of the MLD (SC). The first plethysmography measurement was performed after a period of accommodation of 30 min. Measurements were made three times to obtain baseline values. Then, the animal was gently removed from the chamber for the subcutaneous injection and replaced in the chamber for another session of respiratory recording. Ventilation was recorded during 1 min 30 at each different recording times of plethysmography (T5, T10, T15, T20, T30, T45, T60, T90, T120, T150, T180 min).

#### 2.5.4. Study 4: Effects of DMPO and DEPO on Whole Blood Cholinesterase Activities

Blood cholinesterase activities were determined in three groups of animals. The control group (*n* = 2) received the solvent of paraoxon compounds (isotonic saline solution, SC). The second group (*n* = 3) received DMPO at 0.250 mg/kg corresponding to 50% of the MLD (SC). The last group (*n* = 3) received DEPO at 0.215 mg/kg corresponding to 50% of the MLD (SC).

Whole blood collections were performed by tail punctures at T0 (before injection of paraoxon compounds), then at different times (T5, T10, T15, T30, T60, T90, T120, T180 min). Blood samples were immediately stored at −80 °C until whole blood cholinesterase activity determinations.

#### 2.5.5. Study 5: Toxicodynetic Assessment of Respiratory and Non-Respiratory Toxicities and Whole Blood Cholinesterase Inhibition

The time-course of toxic effects was described by the method reported by Baud et al. [21]. Time of injection of OPs was T0 of clinical and biological effects. In all our experiments dealing with respiratory toxicity of cholinesterase inhibitors, the time-course of effects (toxicodynetics) could be described by a four-phase model centered around the maximal effect (Emax). In our studies, non-lethal toxicity is well described by three major effects: the respiratory rate, the hypothermia, and the inhibition of cholinesterases. Accordingly, we described the toxicodynetics of these two parameters using the following parameters: maximal alteration of respiratory rate as Emax^RR^, minimal value of core temperature (Emax^CTemp^), and minimal residual cholinesterase activity defined as Emax^ChEinh^. Having defined these Emax, we determined (i) the delay in onset of each Emax, (ii) the time of occurrence of these Emax (Tmax^RR^, Tmax^CTemp^, and Tmax^ChEinh^), (iii) the pattern of decrease in these Emax that may be described by a peak or a plateau of effect, and (iv) the delay returning to normal values [21].

### 2.6. Statistical Analysis

#### 2.6.1. Study 2: Rats’ Core Temperature Measurement

Results are expressed as mean ± SEM (standard error of the mean). Three groups of six animals were used. Groups were compared using a two-way analysis of variance for repeated measurements using Prism version 5.0, GraphPad Software (San Diego, CA, USA).

For each parameter, a significant treatment multiplied by time interaction was found (*p* < 0.001); this analysis was followed by multiple pairwise comparisons, the global risk being fixed at *p* < 0.05. All tests were two-tailed.

#### 2.6.2. Study 3: Plethysmography Study of the Effects of DMPO and DEPO on Ventilation at Rest in Rats

Three groups of six animals were used. The same statistical analysis was performed as reported previously.

#### 2.6.3. Study 4: Effects of DMPO and DEPO on Whole Blood Cholinesterase Activities

Values of cholinesterase activities in the different groups were compared using a two-way analysis of variance for repeated measurements as stated above.

## 3. Results

### 3.1. Study 1: Determination of the Median Lethal Doses (MLDs) of DMPO in the Rats

MLD of DMPO by subcutaneous administration was 0.50 mg/kg. Therefore, in the following experiments, a 0.25 mg/kg dose equal to 50% of MLD was used. The MLD of DEPO was previously measured at 0.43 mg/kg [11].

### 3.2. Study 2: Rats’ Core Temperature Measurement

At T0, there were no significant differences in core temperature between control and DMPO and DEPO groups. Following the injection of 0.250 mg/kg of dimethyl paraoxon, a significant decrease (*p* < 0.01 and *p* < 0.001) in the core temperature was observed from T10 to T30. Minimal temperature value of 36.4 ± 0.2 °C in the dimethyl paraoxon group versus 37.5 ± 0.15° C in the control group was obtained to T30. After T60, hypothermia spontaneously corrected and temperatures were not different from control group from T90 to T180.

**Figure 2:**
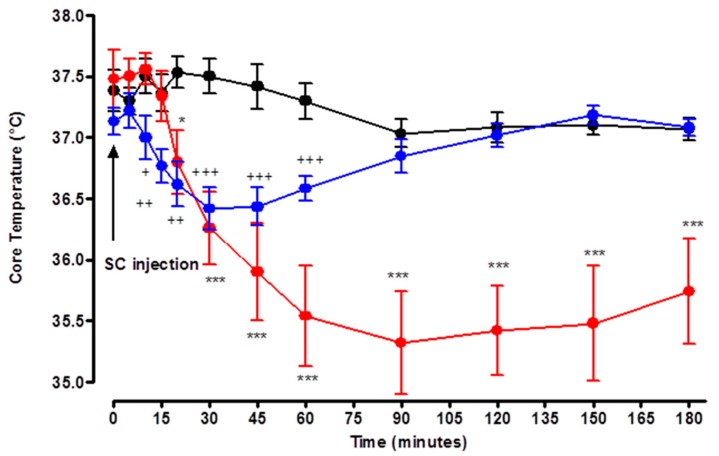
Effects of solvent (black circle), DMPO 50% median lethal dose (MLD50) (blue circle), and diethyl paraoxon (DEPO) 50% MLD50 (red circle) on core temperature. Arrow denotes the time of injection of solvent, DMPO or DEPO. Each group consisted of six rats. Data are represented by mean ± SEM at each time after injection. DMPO vs. control: + *p* < 0.05, ++ *p* < 0.01, and +++ *p* < 0.001. DEPO vs. control: * *p* < 0.05, ** *p* < 0.01, and *** *p* < 0.001.

Following the injection of 0.215 mg/kg of DEPO, a significant decrease (*p* < 0.001) in the core temperature was recorded 30 min post-injection. Minimal value of 35.7 ± 0.5 °C in the DMPO group versus 37.5 ± 0.15 °C in the control group (*p* < 0.001) was recorded at T90 and remained significantly lower than the control group until the end of the study period (Figure 2).

### 3.3. Study 3: Plethysmography Study of the Effects of DMPO and DEPO on Ventilation at Rest in Rats

#### 3.3.1. Comparison of Baseline Values

There were no significant differences in respiratory parameters (f, TE, TI, TTOT, VT, and VE) between control and DMPO and DEPO groups.

#### 3.3.2. Clinical Findings

The animals in the control group were free of symptoms. In the treated group, the most frequent clinical abnormalities were shivering, drooling, prostration, as well as excessive urination and defecation.

In DMPO-poisoned rats, clinical signs appeared at T5 and totally disappeared at T60 min post-injection.

In DEPO-poisoned rats, clinical signs appeared at T10 and persisted until the end of the observation period.

#### 3.3.3. Whole Body Plethysmography

**Figure 3:**
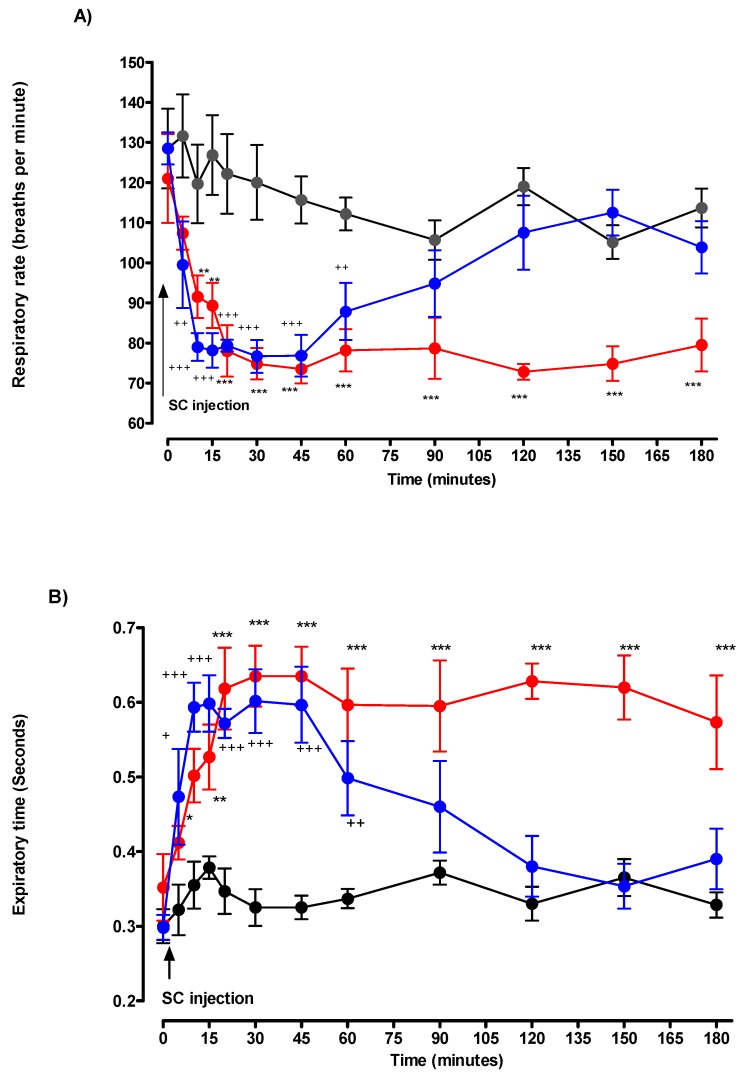
Effects of solvent (black circle), DMPO 50% MLD50 (blue circle), and DEPO 50% MLD50 (red circle) on the (**A**) respiratory rate and (**B**) expiratory time. Arrow denotes the time of injection of solvent, DMPO or DEPO. Each group consisted of six rats. Data are represented by mean ± SEM at each time after injection. DMPO vs. control: + *p* < 0.05, ++ *p* < 0.01, and +++ *p* < 0.001. DEPO vs. control: * *p* < 0.05, ** *p* < 0.01, and *** *p* < 0.001.

Following the injection of 0.250 mg/kg of DMPO, f values were significantly decreased from T5 to T45. Maximum decrease was observed at T10: 78 ± 4 breaths/min in the DMPO group versus 116 ± 6 breaths/min in the control group (*p* < 0.001). After T45, a gradual increase in respiratory rate was observed until T120. From T120 to the end of the experiment; thereafter, f values in the DMPO group remained stable and comparable with the control group (Figure 3).

**Figure 4:**
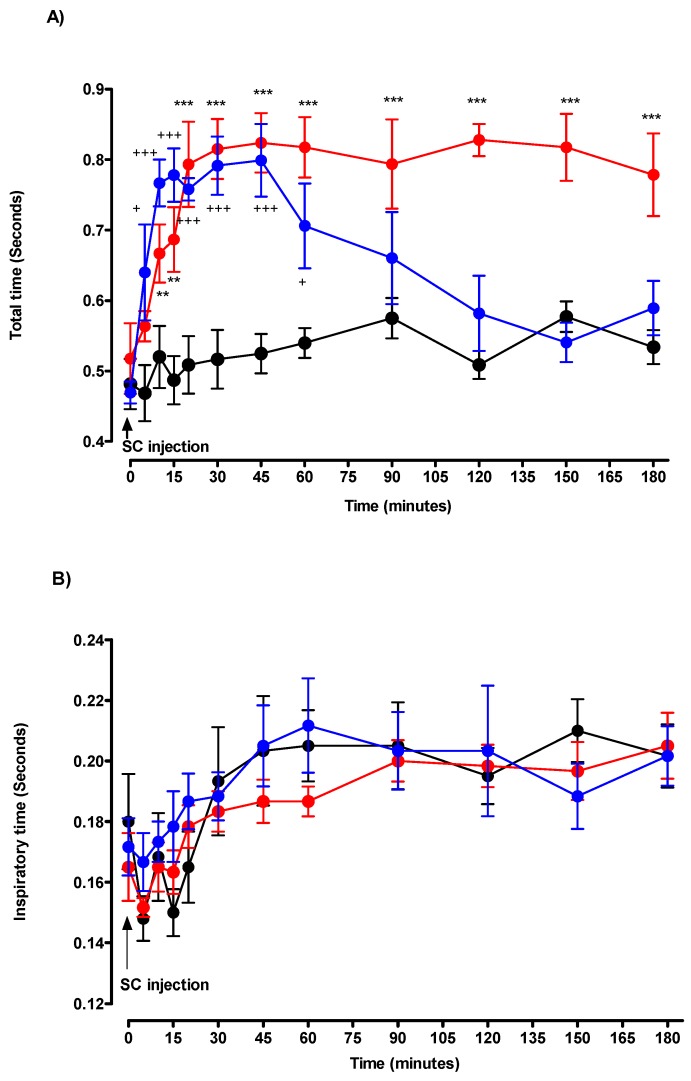
Effects of solvent (black circle), DMPO 50% MLD50 (blue circle), and DEPO 50% MLD50 (red circle) on (**A**) total time, and (**B**) inspiratory time. Arrow denotes the time of injection of solvent, DMPO, or DEPO. Each group consisted of six rats. Data are represented by mean ± SEM at each time after injection. DMPO vs. control: + *p* < 0.05, ++ *p* < 0.01, and +++ *p* < 0.001. DEPO vs. control: * *p* < 0.05, ** *p* < 0.01, and *** *p* < 0.001.

Decrease in respiratory rate was due to a significant increase in TE in comparison with the control group. Maximal values for TE occurred at T10: 0.59 ± 0.03 s versus control: 0.36 ± 0.03 s (*p* < 0.001). Expiratory time values were significantly increased from T10 to T60. From T75 to T120, TE values remained stable and comparable with the control group (Figure 3). After DMPO injection, TTOT values significantly increased from T10 to T45 compared to the control group. The increase in TTOT resulted from an increase in TE with no significant effect on TI. From T75 to T120, TTOT values remained stable and comparable with the control group (Figure 4).

**Figure 5:**
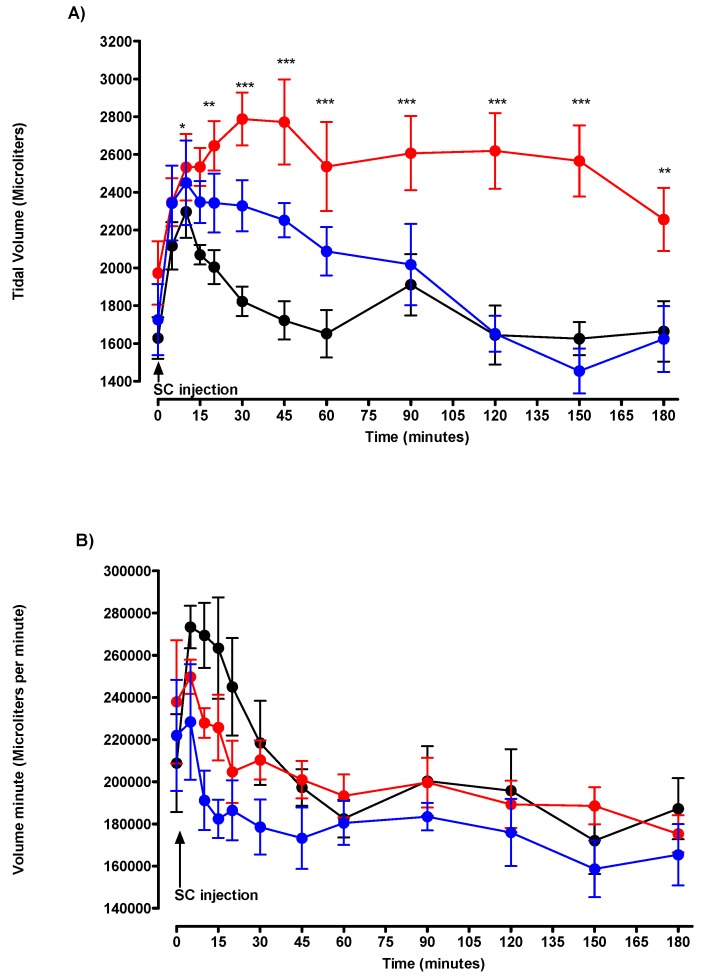
Effects of solvent (black circle), DMPO 50% MLD50 (blue circle), and DEPO 50% MLD50 (red circle) on the (**A**) tidal volume and the (**B**) volume minute. Arrow denotes the time of injection of solvent, DMPO, or DEPO. Each group consisted of six rats. Data are represented by mean ± SEM at each time after injection. DMPO vs. control: + *p* < 0.05, ++ *p* < 0.01, and +++ *p* < 0.001. DEPO vs. control: * *p* < 0.05, ** *p* < 0.01, and *** *p* < 0.001.

Regarding respiratory volumes, the 0.250 mg/kg of DMPO induced a transient, from T5 to T60, but not significant increase in VT values. DMPO injection was without effect on VE (Figure 5).

Following the injection of 0.215 mg/kg of DEPO, f values were significantly decreased from T0 to T15 (*p* < 0.01). Maximum decrease was observed at T30: 74 ± 4 breaths/min versus 116 ± 6 breaths/min (*p* < 0.001). For DEPO, respiratory rate remained low and significantly different from control until the end of the experiment (Figure 3).

Conversely, in comparison with the control group, the TE values were significantly increased in the DEPO-poisoned group from T10. Maximal values for TE occurred to T30: 0.64 ± 0.04 s vs. 0.32 ± 0.02 s (*p* < 0.001), and the increase remained stable until the end of the study (Figure 3).

After DEPO injection, TTOT values significantly increased from T10 to T180. The increase in TTOT resulted from an increase in TE with no significant effect on TI (Figure 4).

Regarding respiratory volumes, the 0.215 mg/kg of DEPO, induced a significant increase in VT values from T15 to the end of the experiment. DEPO injection was without effect on VE (Figure 5).

### 3.4. Study 4: Effects of DMPO and DEPO on Whole Blood Cholinesterase Activities

**Figure 6:**
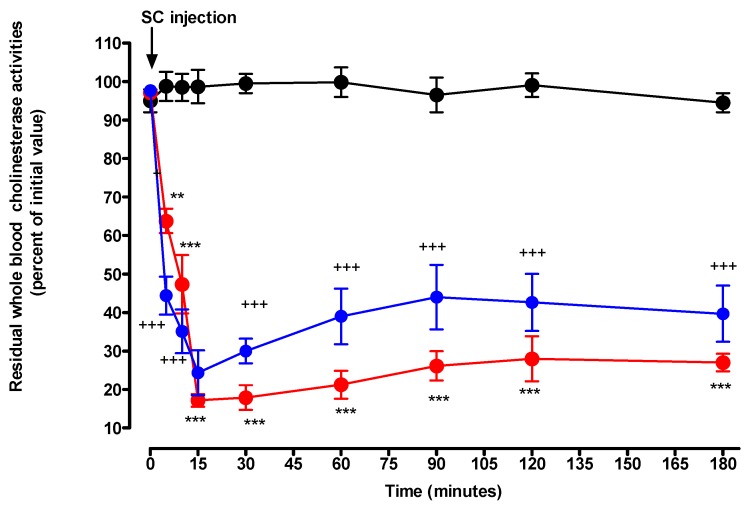
Time-courses of residual whole blood cholinesterase activities in control (black circle), DMPO 50% MLD50 (blue circle), and DEPO 50% MLD50 (red circle) treated groups. Control group consisted of two rats and treated groups of three rats. Arrow denotes the time of injection of solvent, DMPO, or DEPO. Data are represented by mean ± SEM at each time after injection. DMPO vs. control: + *p* < 0.05, ++ *p* < 0.01, and +++ *p* < 0.001. DEPO vs. control: * *p* < 0.05, ** *p* < 0.01, and *** *p* < 0.001.

After injection of dimethyl or diethyl paraoxon at 50% MLD doses, significant decreases in whole blood cholinesterase activities were observed from T5 to T180. For DMPO and DEPO, maximal inhibitions occurred to T15. At that time, residual activities were 24 ± 6% and 17 ± 5%, after injection of DMPO and DEPO, respectively. For DMPO, a slight but insignificant increase in whole blood cholinesterase activities was observed from T30 to T180, ranging from 30 to 40% for residual cholinesterase activities. In the DEPO group, residual activities stayed low until the end of the experiment with a slight but insignificant increase in values from T30 to T180. There were no significant differences between residual whole blood cholinesterase activities between DMPO and DEPO groups at any time (Figure 6).

### 3.5. Study 5: Toxicodynetic Assessment of Respiratory and Non-Respiratory Toxicities and Whole Blood Cholinesterase Inhibition

The life-threatening of clinical and biological effects (Emax^RR^, Emax^CTemp^, and Emax^ChEinh^) resulted from respiratory toxicity. The most striking effects induced by DEPO and DMPO were increased in expiratory time. Experimental studies showed the absence of any lag-time for the onset of both DMPO and DEPO as the TE started to increase as soon as 5 min post-injection. Thereafter, there was a rapid and linear worsening resulting in reaching the Emax^RR^ of increase in TE between 20 and 30 min after injection of both DMPO and DEPO. Noteworthy, equipotent dose of each induced an Emax^RR^ of the same magnitude. Thereafter, there was a sharp discrepancy in the time-course of Emax^RR^ induced by the two paraoxon derivatives.

DMPO induced a short duration of Emax^RR^ plateau which lasted 30 min followed by a linear decrease, spontaneously returning to values not significantly different from the normal values as soon as 90 min post-injection, corresponding to 15 min after the end of the plateau. Within the 180 min period of observation, there was no evidence of rebound of toxicity induced by DMPO. The TE values induced by DMPO were not significantly different from control values during 90 min corresponding to half the duration of observation of the animals.

In contrast, DEPO induced a long-lasting plateau of Emax^RR^ values that were still significantly greater than the control values at 180 min post-injection with no trend toward a progressive decline. Therefore, owing to the duration of observation, we were unable to describe the late phase of the toxicodynetics of DEPO.

One significant clinical effect was PO-induced hypothermia for which an Emax^CTemp^ of hypothermia could be described. Even in the early phases, the toxicodynetics of hypothermia induced by DEPO and DMPO were different. DEPO induced a 5 min delay in onset of hypothermia. In contrast, DMPO induced a 10 min delay in onset of hypothermia.

The Emax^CTemp^ of hypothermia induced by DMPO was significantly lower than that induced by DEPO. DMPO-induced Emax^CTemp^ on hypothermia occurred at 30 min post-injection, whereas this delay was 90 min after DEPO injection.

There was a short, 10 min, plateau of Emax^CTemp^ on hypothermia-induced DMPO. In contrast, the plateau of Emax^CTemp^ induced by DEPO was long-lasting and persisted at the end of the 180 min duration of observation.

In contrast with the major differences in the toxicodynetics of TE and hypothermia between DMPO and DEPO, the magnitude and duration (Emax^ChEinh^) of inhibition of whole blood cholinesterase were strongly similar (Figure 6). There was no delay in onset on inhibition with an extremely rapid linear worsening of inhibition with an Emax^CTemp^ of hypothermia occurring 15 min post-injection and lasting 180 min post-injection with no evident trend toward improvement.

## 4. Discussion

There are numerous publications about cases of humans poisoned by OP insecticides [2,4,5]. Whatever the conditions, accidental or self-inflicted, OP poisoning is a potentially life-threatening intoxication [22]. Therefore, supportive and specific treatments should be administered as soon as possible and as long as necessary. Consequently, the whole course of poisoning without treatment is not known in humans. Only experimental animal models have given insight into one of the spontaneous courses of signs and symptoms in a non-lethal as well as a lethal model [12]. In an experimental model, Rickett et al. [23] were able to determine the different mechanisms of acute respiratory arrest induced by chemical warfare agents.

There is limited knowledge about the time-course of OP poisonings in humans. Although the mechanisms of toxicity are thought to be the same for all organophosphate insecticides, the delay in onset of the early phase related to the class of OPs in terms of oxon and thion chemical structures is well known. The intermediate phase induced by OPs has also been clearly described [24]. A limited number of agents were shown to induce delayed polyneuropathy [25]. Finally, the occurrence of aging was shown to strongly depend on the nature of alkoxy groups that may dramatically alter the efficacy of oximes in OP-poisoned animals and humans [26]. Eddleston et al. [22] reported that, among 802 patients poisoned with chlorpyrifos, dimethoate, or fenthion, differences between the chemistry of OP pesticides accounted for differential toxicity and different efficacy of antidotal treatment. They noted that the relative human toxicity of these insecticides might not be related to animal toxicity. They also noted that the widely used approach of differentiating organophosphorus insecticides according to their animal LD50 was not in agreement with human toxicity and was probably of limited value in risk assessment and management of acute human poisoning.

The absence of knowledge about the spontaneous course of human poisonings induced by each chemical agent may account for the apparent great variability in toxicity and might be a major limitation to assess the actual efficiency of treatment. The in vitro experimental approach provides highly valuable information on toxic effects at a selected time but does not allow assessing the whole course of the poisoning. This limitation of in vitro studies encouraged us to develop a non-lethal model in awake rats focusing on respiratory toxicity which is the main target of OPs [11,16]. Furthermore, we are not aware of any temporal systematization of data collection in toxicological studies. To address this major limitation, we developed the concept of toxicodynetics which allows researchers to describe the time-course of effects in animals as well as humans [19]. In contrast with data collected in humans, we observed a highly reproducible respiratory toxicity of a non-lethal dose of diethyl paraoxon in rats [11,13,16,27]. We extended the reproducibility of respiratory toxicity of the same dose of diethyl paraoxon in mice [8].

In the present study, we assessed the respiratory toxicity of two compounds in which the lone difference was dissimilar alkoxy groups, namely methyl or ethyl. Noteworthy, both compounds exhibit the same magnitude of log *P* values [19]. Furthermore, in the present study for DMPO and a previous study for DEPO [11], MLDs were rather similar, 0.50 and 0.43 mg/kg, respectively.

Using the same equipotent doses of DMPO and DEPO, the toxicodynetic approach showed similar early phases, including the absence of delay in onset, the rapid worsening of the TE which occurred at the same Tmax, with the same magnitude of Emax^RR^. In contrast, there was a drastic difference in the late phase including the short duration of the plateau induced by DMPO contrasting with the long-lasting one induced by DEPO. Actually, the duration of our observations allowed us to describe the spontaneous reversal of DMPO respiratory toxicity. However, we were unable to describe the complete recovery from DEPO respiratory toxicity.

We previously reported the same pattern of respiratory toxicity using nearly equipotent doses of dichlorvos [19], in comparison with DMPO. Indeed, the toxicodynetics of dichlorvos, a dimethyl-derivative, showed short-lasting respiratory toxicity with an Emax^RR^ of TE of the same order of magnitude than that induced by DMPO, followed by rapid spontaneous normalization [19]. This short-lasting duration of respiratory toxicity was unexpected owing to the more rapid aging of dimethyl-derivatives in contrast with diethyl compounds. Conversely, the short duration of respiratory toxicity may question the need of oximes to treat this group of OPs owing to the efficiency of atropine.

The differences in toxicodynetics of DMPO and DEPO contrast with the long-lasting plateauing inhibition of whole blood cholinesterase activities. Noteworthy, the magnitude of inhibition was similar for DMPO and DEPO, 70 and 80 percent, respectively. There was only a slight difference in the inhibitory effect between of these two compounds. A similar difference was also observed during reactivation by oximes of total cholinesterases inhibited by dimethyl and diethyl compounds [28]. In a previous study, we were able to assess the toxicokinetic-toxicodynamic (TK/TD) correlation for the dose of DEPO that was used in the present study [18]. The TK/TD study showed the complete, albeit transient, efficacy of pralidoxime in reversal DEPO-induced respiratory toxicity for 30 min followed by a rebound of respiratory toxicity at the pre-treatment level. This short duration of action of pralidoxime as well as the rebound of respiratory toxicity contrasted with long-lasting nearly complete whole blood cholinesterase reactivation. In our study comparing respiratory toxicity induced by DMPO and DEPO assessed without pralidoxime treatment, we observed a major discrepancy with long-lasting whole blood cholinesterase inactivation in animals poisoned by both compounds, where DMPO showed short-lasting respiratory toxicity and, at the opposite end, DEPO induced long-lasting respiratory toxicity.

Our study has a number of limitations. Many organophosphorus compounds, including dichlorvos, chlorpyrifos, and fenthion, exist as dimethyl or diethyl forms as does paraoxon. We tested only one OP, paraoxon. Additional alkoxy- and plain phosphate-bearing compounds must be included for the conclusions to be considered solid. Conclusions based on two compounds are too broad. Therefore, we cannot extend our findings to other compounds. The duration of the study was too short to describe the complete course of DEPO. Cholinesterase activities were determined only in whole blood but not in targeted tissues.

## 5. Conclusions

Equipotent doses of DMPO and DEPO induced the same magnitude of whole blood cholinesterase inhibition. In contrast, the toxicodynetics were markedly different with the same early phases but a strongly different late phase and recovery. These findings suggest that the nature of the alkoxy group can be a major determinant for the toxicodynetics of respiratory effects, whereas the chemical structure corresponding to phosphorus ester can be a major determinant for whole blood cholinesterase inhibition. These data support the hypothesis of a complex correlation between whole blood cholinesterase inhibition and OP-induced respiratory toxicity.
